# Yeast Derived LysA2 Can Control Bacterial Contamination in Ethanol Fermentation

**DOI:** 10.3390/v10060281

**Published:** 2018-05-24

**Authors:** Jun-Seob Kim, M. Angela Daum, Yong-Su Jin, Michael J. Miller

**Affiliations:** 1 Department of Food Science and Human Nutrition, University of Illinois, 905 S. Goodwin Ave., Urbana, IL 61801, USA; junseob.kim.83@gmail.com (J.-S.K.); adaum@farmhouseculture.com (M.A.D.); ysjin@illinois.edu (Y.-S.J.); 2 Carl R. Woese Institute for Genomic Biology, University of Illinois at Urbana-Champaign, 1206 W. Gregory Dr., Urbana, IL 61801, USA

**Keywords:** endolysin, *Saccharomyces cerevisiae*, biofuel, *Lactobacillus fermentum*, fermentation, *Pichia pastoris*, secretion, endopeptidase, sugarcane

## Abstract

Contamination of fuel-ethanol fermentations continues to be a significant problem for the corn and sugarcane-based ethanol industries. In particular, members of the *Lactobacillaceae* family are the primary bacteria of concern. Currently, antibiotics and acid washing are two major means of controlling contaminants. However, antibiotic use could lead to increased antibiotic resistance, and the acid wash step stresses the fermenting yeast and has limited effectiveness. Bacteriophage endolysins such as LysA2 are lytic enzymes with the potential to contribute as antimicrobials to the fuel ethanol industries. Our goal was to evaluate the potential of yeast-derived LysA2 as a means of controlling *Lactobacillaceae* contamination. LysA2 intracellularly produced by *Pichia pastoris* showed activity comparable to *Escherichia coli* produced LysA2. Lactic Acid Bacteria (LAB) with the A4α peptidoglycan chemotype (L-Lys-D-Asp crosslinkage) were the most sensitive to LysA2, though a few from that chemotype were insensitive. *Pichia*-expressed LysA2, both secreted and intracellularly produced, successfully improved ethanol productivity and yields in glucose (YPD60) and sucrose-based (sugarcane juice) ethanol fermentations in the presence of a LysA2 susceptible LAB contaminant. LysA2 secreting *Sacharomyces cerevisiae* did not notably improve production in sugarcane juice, but it did control bacterial contamination during fermentation in YPD60. Secretion of LysA2 by the fermenting yeast, or adding it in purified form, are promising alternative tools to control LAB contamination during ethanol fermentation. Endolysins with much broader lytic spectrums than LysA2 could supplement or replace the currently used antibiotics or the acidic wash.

## 1. Introduction

In the search for renewable sources to replace petroleum-based fuel, ethanol from carbohydrate-rich plant sources continues to outstrip the alternatives in terms of production and efficiency at the desired scales. The United States of America (US) and Brazil combined produced more than 20 billion gallons of fuel ethanol primarily from corn and sugarcane in 2014 [1]. Among the various outstanding issues, bacterial contamination of fuel ethanol fermentation continues to be a challenge to producers [2,3,4,5], and can make the difference between running at a profit or a loss [6]. For example, one analysis determined that a 100 MMgy fuel ethanol plant with a moderate level of contamination can expect an annual revenue loss of $4.5 million at 2016 ethanol prices [7].

Both wild yeasts and bacteria that thrive under the ethanol fermentation conditions preferred by industrial *Saccharomyces cerevisiae* strains have been identified as sources of contamination. The predominant bacterial contaminants are Gram positive bacteria from the *Lactobacillaceae* family, including *Lactobacillus*, *Pediococcus*, *Leuconostoc*, *Enterococcus*, *Aerococcus*, and *Weisella* [3,5,6,8]. These bacteria, which produce primarily lactic acid from carbohydrate sources, are often referred to as Lactic Acid Bacteria (LAB), and many have adapted to conditions with comparatively low pHs and oxygen levels, as well as high salt and ethanol concentrations. Consequently, LAB contaminations have been a recurring problem in wine, beer, and spirit fermentations [9,10,11]. Biofuel fermentations are particularly at risk from LAB contamination because the plants that provide the fermentation substrate are the natural habitats of LAB, and these substrates are rarely subjected to bacteriostatic or bactericidal treatment. The predominant genera of LAB contaminants identified in biofuel fermentations is *Lactobacillus,* regardless of whether the fermentation is from corn [4,8], sugarcane [5,12], or lignocellulose [13]. Treatments to control bacterial contamination of fuel ethanol fermentation must include measures against lactobacilli and other contaminating LAB, in order to be effective. Methods explored to combat contamination by LAB include pretreatment of feedstock, changing process conditions such as solids content and pH, cleaning fermentation equipment, antibiotics, bacteriocins, acid washing of yeast used for sugarcane fermentations, and bacteriophages. Currently, acid treatment of the yeast, cleaning of equipment, and antibiotics are the most commonly used measures in industry. Acid-washing stresses the fermenting yeast and can lead to decreased ethanol production efficiency [14]. US fuel ethanol plants use penicillin and virginiamycin [15,16] while Brazilian plants are more likely to use monensin [17,18] although the efficacy of antibiotic treatments has been challenged [19]. Additional complications from antibiotic use are unacceptably high antibiotic residues in co-products, the dried distillers grain solids from corn ethanol plants [20] or dried deactivated yeasts [2] from sugarcane ethanol plants. Increased development of antibiotic resistance in the contaminating strains has been noted as well [19].

Alternative approaches to control the LAB are needed. Researchers are exploring the efficacy of bacteriophages (phages) [4], and in specific instances, have applied for patents using phages as an antimicrobial strategy [21]. The use of phages is an interesting approach, but has a number of drawbacks, including the specificity of the phage to a particular strain and the numerous adaptation mechanisms bacteria have developed, and continue to develop, to evade phage infection. However, phage lysins, proteins which allow the phages to burst bacterial cells, can be applied externally to lyse Gram positive bacteria [22,23,24]. Some lysins, including LysA2, have been shown to impact multiple different strains of LAB, and could be useful in reducing fuel ethanol contamination [25,26]. Lysins have several potential advantages, including an appropriate lytic spectrum that allows individual lysins to lyse several genera of LAB [26]. In addition, lysins have a reduced potential for resistance development compared to antibiotics [27,28]. Lastly, it is possible that the fermenting yeast could heterologously produce the desired lysin at a lower cost.

LysA2 is an endopeptidase from the *Lactobacillus casei* phage Ø393 A2 [25]. *Lactobacillus casei* phage Ø393 A2 was isolated from the whey of a failed fermentation of Gamoneu blue cheese [29]. Previous studies found that LysA2 decreased the optical density, as measured by absorbance readings at 600 nm (OD) of a number of LAB [25,26]. These studies suggest that LysA2 might be effective in inhibiting the unwanted growth of LAB in biofuel fermentations. This study further explores the spectrum and activity of LysA2 produced by the yeasts *Pichia pastoris* GS115 and *Saccharomyces cerevisiae* D452-2. To our knowledge, this is the first time that a *P. pastoris* or *S. cerevisiae* secreting LysA2, or any other endolysin, has been reported in the literature.

## 2. Materials and Methods

### 2.1. Bacterial and Yeast Strains

All bacterial and yeast strains used in this study are listed in Table 1. *E. coli* were cultured in lysogeny broth (LB) and incubated aerobically at 37 °C. When necessary, LB was supplemented with 100 μg/mL ampicillin. Yeast were cultured in YPD medium (1% yeast extract, 2% peptone and 2% glucose), and incubated aerobically/anaerobically (5% CO_2_, 5% H_2_ and 90% N_2_) at 30°C.

### 2.2. Construction of a LysA2 Bacterial and Yeast Expression Vectors

Bacterial and yeast expression vectors for LysA2 were constructed based on Invitrogen plasmids (Figure S1). The entire LysA2 open reading frame was codon optimized for *E. coli* BL21 and *P. pastoris* GS115. The *E. coli* optimized LysA2 gene was cloned into a pRSET A expression vector (pRSETA_LysA2). The *P. pastoris* optimized LysA2 gene was cloned into pPICZA intracellular (pPICZA_LysA2) and pPICZα (pPICZAa_LysA2) secretion expression vectors. The *S. cerevisiae* D452-2 strain was used for LysA2 secretion and genome integration. The *P. pastoris* optimized LysA2 gene with α mating factor was PCR amplified from pPICZAa_LysA2, and cloned into pRS423 expression vector containing the GPD promoter and CYC1 terminator for *S. cerevisiae* secreted expression (pRS423_LysA2). For integration into the *S. cerevisiae* genome, the plasmid pITy3 (pITY3_LysA2) was employed, as previously described [30].

### 2.3. Bacterial and Yeast Transformation with the LysA2 Bacterial and Yeast Expression Vectors

*E. coli* cells were cultured overnight in 6 mL LB and harvested by centrifugation. Cellular pellets were washed four times in 1 mL 300 mM sucrose, and collected by centrifugation. After washing, the cellular pellet was resuspended in 100 μL of 300 mM sucrose, and used for electroporation at 2.5 kV with pRSETA_LysA2. Putative *E. coli* transformants were selected by ampicillin-containing LB agar plates (100 μg/mL).

*P. pastoris* cells were cultured in YPD medium until reaching an OD_600_ of 2.0. Approximately 8 × 10^8^ cells were suspended in 8 mL of P-transformation buffer (100 mM lithium acetate, 10 mM dithiothreitol, 0.6 M sorbitol and 10 mM Tris-HCL, pH 7.5) for 30 min at room temperature. *P. pastoris* cells were harvested and washed three times in 1.5 mL of ice-cold 1 M sorbitol. The cellular pellet was then re-suspended in 100 μL of ice-cold 1 M sorbitol and used for electroporation (1.5 kV) with pPICZA_LysA2 (intracellular) or pPICZα_LysA2 (secretion). Putative *P. pastoris* transformants were screened using agar plates containing 2000 μg/mL of Zeocin.

pRS423_LysA2 and pITY3_LysA2 were transformed into *S. cerevisiae* using the high-efficiency yeast transformation protocol [31]. Positive transformants were selected via the appropriate auxotrophic marker, using yeast synthetic complete medium, as described by Jin et al. [32].

### 2.4. Protein Expression and Purification

*E. coli* cells containing pRSETA_LysA2 were cultured overnight at 37 °C in LB with 100 μg/mL ampicillin. Cell cultures were passaged (1%, *v*/*v*) into fresh LB and incubated at 37 °C with agitation at 200 rpm until an OD_600_ of 0.5. To induce protein expression, 0.2 mM IPTG was added, with further incubation for 5 h at 30 °C with agitation at 200 rpm.

*P. pastoris* cells containing pPICZA_LysA2 or pPICZα_LysA2 were cultured overnight at 30 °C in BMGY-Buffered Glycerol-complex medium, and harvested by centrifugation (4000 rpm at 4 °C). *P. pastoris* cell pellets were resuspended to an OD_600_ of 1.0 in BMMY-Buffered Methanol-complex medium. To induce protein expression, methanol was added every 24 h.

After the appropriate incubation, induced cell cultures were washed twice in binding buffer (300 mM NaCl, 20 mM imidazole, 10% glycerol in 50 mM phosphate buffer, pH 7.5). Cells were broken using a French press cell homogenizer five times, and centrifuged at 20000 ×g for 20 min at 4°C to remove insoluble contents. Soluble lysate was added to a column containing Ni-NTA beads (His60 Ni Superflow Resin, Clontech, Moutain View, CA, USA), and incubated for 3 h at 4°C. The column was washed with 10 column volumes of washing buffer (300 mM NaCl, 40 mM imidazole, 10% glycerol in 50 mM phosphate buffer, pH 7.5). His-tagged LysA2 protein was eluted with elution buffer (300 mM NaCl, 300 mM imidazole, 10% glycerol in 50 mM phosphate buffer, pH 5.5). Purified LysA2 proteins were confirmed by SDS-PAGE using standard procedures. For evaluation of secreted LysA2 from *P. pastoris*, the culture medium from the induced cells described above was collected by centrifugation. When indicated, the culture medium was concentrated 50 fold using an Amicon ultra centrifugal filter. For evaluation of secreted LysA2 production by *S. cerevisiae* (both plasmid and chromosomally encoded LysA2 strains), the supernatant of *S. cerevisiae* cultures at 24 h were taken and concentrated 20 times using an Amicon ultra centrifugal filter, and analyzed by SDS-PAGE (Figure S2). Two target bands were identified and isolated from the protein gel, and analyzed by LC/MS after trypsin treatment. The sequencing results were analyzed using a Mascot distiller and Mascot search engine against the entire NCBI database, confirming that both bands were indeed LysA2.

### 2.5. Turbidity Reduction Assay

Lactic acid bacteria representative of common biofuel contaminants are listed in Table 2. All LAB were cultured in de Man, Rogosa and Sharpe (MRS) broth and incubated at 37 °C anaerobically overnight. Cells for the turbidy reduction assay were prepared as described by Becker and colleagues [22]. Briefly, LAB overnight cells were centrifuged at 13,000 rpm at 4 °C for 1 h. The supernatant was discarded, and the LAB cellular pellets were stored at −20 °C until use. Prior to the turbidity reduction assay, the LAB cellular pellets were thawed and resuspended in 50 nM phosphate buffer, pH 5.5 to an OD ≈ 2. Purified LysA2 produced by *P. pastoris* was mixed with the phosphate buffered LAB suspensions at 12.5, 25, 50, 100, 1000 nM in a final volume of 100 μL. The reduction in turbidity at 595 nm was measured using a microplate reader (Multiskan Ascent, Thermo Fisher, Waltham, MA, USA).

Lytic activity of the culture supernatant containing secreted LysA2 from *P. pastoris* was measured, as described above, for the purified LysA2 proteins. When needed, culture supernatant was concentrated using an Amicon ultra centrifugal filter.

### 2.6. Fermentation with Simulated Contamination

Mock fermentations were conducted with YPD containing 60 g/L glucose (YPD60) or clarified sugarcane juice. Clarification of sugarcane juice was completed via centrifugation to remove solid contents and filtration with a 0.4 nm bottle-top filter. Clarified sugarcane juice was diluted with purified water to ~6% total sugars content, and supplemented with 0.6 g/L yeast extract. The addition of yeast extract served to replace dead yeast that is typically present in industrial fermentations in Brazil [33,34,35].

*S. cerevisiae* cells were cultured in synthetic complete glucose broth anaerobically at 30 °C for 24 h, to stabilize the expression vectors. Meanwhile, *L. fermentum* ATCC 9338 and *L. plantarum* ATCC 14917 were cultured in MRS broth anaerobically at 30 °C for 24 h. *S. cerevisiae* and *L. fermentum* ATCC 9338 or *L. plantarum* ATCC 14917 were co-inoculated from their separate cultures at an OD_600nm_ 0.5 and 0.05, respectively for YPD60 and OD_600nm_ 5.0 and 0.5, respectively for sugarcane juice. All mock fermentations were performed in a total of 20 mL, and incubated anaerobically at 30 °C, with agitation at 100 rpm for 36 h. At designated intervals, the OD at 600 nm was measured, and 1 mL samples were collected for high-performance liquid chromatography (HPLC) analysis, as previously described [36].

## 3. Results

### 3.1. Comparison of Lytic Activity of LysA2 Expressed by Bacteria and Yeast

**Figure 1:**
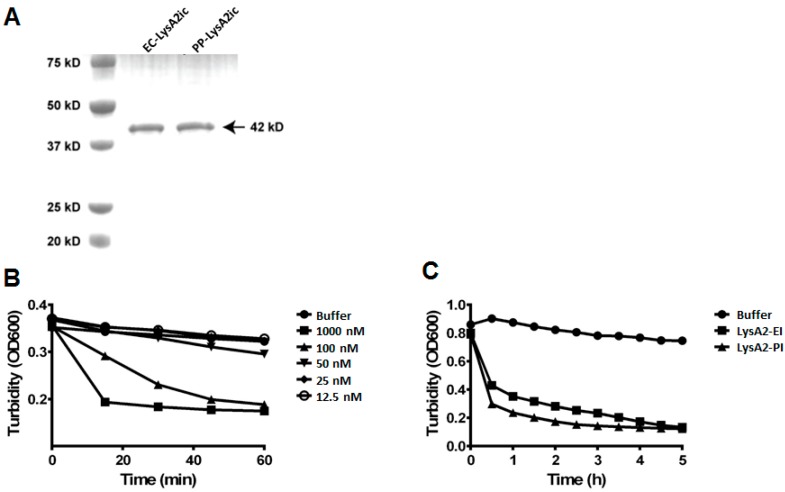
Purification and lytic activity of intracellularly expressed LysA2 from *P. pastoris* and *E. coli*. Intracellularly produced LysA2 from *E. coli* (LysA2-EI) and *P. pastoris* (LysA2-PI) were purified and confirmed by protein size and lytic activity. SDS-PAGE analysis shows LysA2-EI and LysA2-PI are approximately the expected size of 42 kDa (**A**). Lytic activity of LysA2-PI measured by turbidity reduction with *L. casei*. Lytic activity increased proportionally with increased protein concentration (**B**). Lytic activity of LysA2-PI (100 nM, 4.22 μg/mL) was compared with LysA2-EI (100 nM, 4.22 μg/mL) using turbidity reduction with *L. casei* (**C**).

A previous study was unable to get *S. cerevisiae* to secrete LysA2 [23]. Consequently, we wanted to explore LysA2 expression, both intracellular and secreted, in *P. pastoris*, which is a eukaryotic model system for heterologous expression of proteins. To compare the activity of LysA2 produced by yeast with the activity of LysA2 from the *Escherichia coli* BL21 (DE3) expression system used by Ribelles et al. [25], *Pichia pastoris* GS115 was employed. Intracellularly expressed His6-tagged LysA2 proteins from *P. pastoris* and *E. coli* were purified. SDS-PAGE analysis revealed both LysA2 protein bands were the predicted size, approximately 42 kDa (Figure 1A), with no apparent glycosylation of the yeast expressed protein.

To measure the lytic activity of LysA2, the turbidity reduction of a suspension of *L. casei* was measured after application of the enzyme. Intracellularly produced LysA2 from *P. pastoris* showed dose-dependent lytic activity, and required concentrations greater than 50 nM to be detected (Figure 1B). The addition of LysA2 from *E. coli* and *P. pastoris* produced a comparable turbidity reduction of *L. casei* cells over five-hour span at the same dose (Figure 1C). This result indicates that LysA2 can be functionally expressed intracellularly in yeast, and the activity of this *P. pastoris* produced LysA2 is equivalent to that of the *E. coli* produced LysA2.

### 3.2. Lytic Activity of Intracellularly Produced LysA2 from P. pastoris on Common Biofuel Contaminants

Thirteen LAB strains, identified as biofuel contaminants in the literature, including six genera with the majority from *Lactobacillus,* were selected to assess the lytic spectrum of the *P. pastoris* intracellularly produced LysA2. LysA2 showed the strongest lytic activity against *L. casei.* Compared to *L. casei, L. paracasei* (++++), *L. delbrueckii* (+++), *L. fermentum* (++), *L. rhamnosus* (++), *E. faecium* (++), *E. gallinarum* (++) and *L. lactis* (+) showed at least some sensitivity to LysA2 (Table 2). *Aerococcus viridans, L. brevis, L. plantarum, Pediococcus acidilacti* and *P. damnosus* were not susceptible to LysA2. LysA2 produced by *E. coli* had a similar lytic activity and spectrum compared to LysA2 from *P. pastoris* (data not shown). Interestingly, three out of five of the non-susceptible strains have the same peptidoglycan chemo-type as *L. casei* (A4α, L-Lys-D-Asp).

### 3.3. Secreted LysA2 and Its Lytic Activity

**Figure 2:**
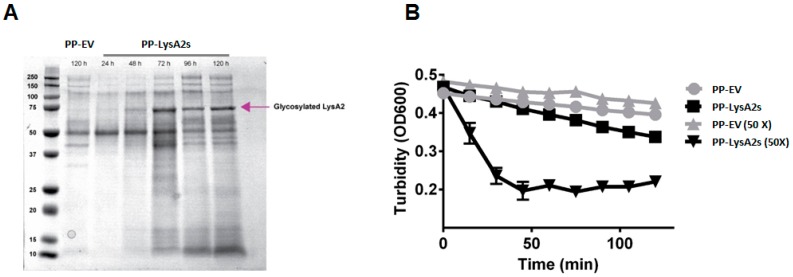
Activity of supernatant containing secreted LysA2 from *P. pastoris.* Secreted LysA2 from *P. pastoris* (PP-LysA2s) was confirmed by SDS-PAGE analysis of the supernatant. The LysA2 in the supernatant increased with increased incubation time. The molecular weight (~72 kDa) was larger than the expected size (42 kDa) which could be due to glycosylation (**A**). Lytic activity was confirmed by turbidity reduction with *L. casei* treated with concentrated (50×) and unconcentrated culture supernatant from the *P. pastoris* secreting LysA2 strain (PP-LysA2s) compared to the culture supernatant from the *P. pastoris* with the empty vector strain (PP-EV) (**B**).

To investigate the lytic activity of LysA2 secreted from yeast, a *P. pastoris* secreting LysA2 strain was constructed. A protein band of approximately 70 kDa was observed in the concentrated culture medium after 1% methanol induction, and a subsequent 48 h of growth (Figure 2A). There was no comparable band from the concentrated culture medium from *P. pastoris* containing the empty vector. This putative LysA2 band had a higher molecular weight than the expected protein size of 42 kDa, and could not be purified using a Ni-NTA column. To ensure the putative secreted LysA2 protein was effective at lysing target bacteria, post induction (72 h) culture medium from the LysA2 secreting *P. pastoris* or from the empty vector *P. pastoris* was applied to *L. casei* cells. Turbidity of the *L. casei* cell suspension only decreased after application of the culture medium from LysA2 expressing *P. pastoris* (Figure 2B). However, a higher concentration of LysA2 from the culture medium (Figure 2B) was necessary to achieve activity comparable to the 100 nM (4.22 μg/mL) of purified LysA2 that was intracellularly produced by *P. pastoris* (Figure 1B), indicating that the secretion level of LysA2 is low, but that the protein is active.

**Figure 3:**
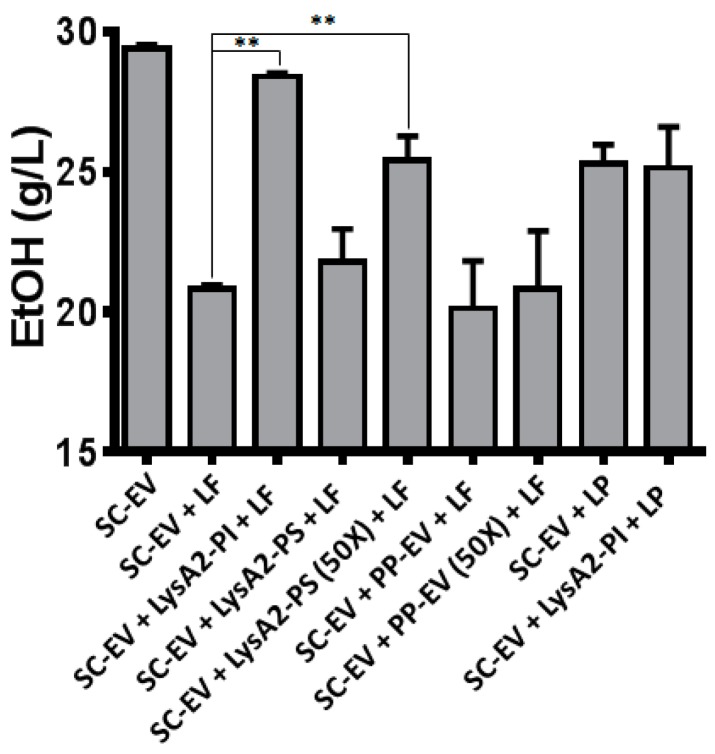
Activity of secreted LysA2 from *P. pastoris* compared to purified intracellularly produced LysA2 from *P. pastoris* in YPD60 fermentations by *S. cerevisiae* D-452 with an empty vector (SC-EV). In the chart above, lane 1 contains SC-EV only, lanes 2–7 are SC-EV and *L. fermentum* (LF) and lanes 8–9 contains SC-EV and *L. plantarum* (LP)*.* The conditions are: (1) no treatment, no contamination; (2) no treatment; (3) purified intracellular LysA2 (LysA2-PI; 100 nM); (4) Supernatant from LysA2 secreting *P. pastoris* (LysA2-PS); (5) Concentrated (50×) supernatant from LysA2 secreting *P. pastoris* (LysA2-PS); (6) Supernatant from *P. pastoris* with empty secretion vector (PS-EV); (7) Concentrated supernatant from *P. pastoris* with empty secretion vector (PS-EV); and (8) no treatment; (9) purified intracellular LysA2 (LysA2-PI; 100 nM). In a fermentation by *S. cerevisiae* empty vector (SC-EV) in YPD60, adding the contaminants *L. fermentum* and *L. plantarum* decreased ethanol compared to the uncontaminated control. Treatment with purified LysA2 produced intracellularly (LysA2-PI) and concentrated or unconcentrated supernatant from *P. pastoris* secreting LysA2 (LysA2-PS) restored ethanol production in contaminations with *L. fermentum* but not with *L. plantarum*. *p* values (** *p* < 0.01) were determined using *t*- test.

Further analysis of the activity of the yeast expressed LysA2 entailed evaluating the impact of LysA2, both intracellular and secreted, on ethanol production by *S. cerevisiae* in a simulated contamination. Frequently identified LAB contaminants *L. fermentum* (LF) or *L. plantarum* (LP) were co-cultured with *S. cerevisiae* in YPD60 (Figure 3). Without contamination (SC-EV in Figure 3), *S. cerevisiae* consumed all glucose (60 g/L) in YPD60, and produced ethanol (28 g/L) within 24 h. LF contamination resulted in a 25% reduction in ethanol titer (SC-EV + LF in Figure 3). Adding purified, intracellularly-produced LysA2 (100 μg/mL) completely restored ethanol titer (SC-EV + LysA2-PI + LF in Figure 3). *P. pastoris* culture media containing secreted LysA2 slightly improved ethanol titer, and the 50-fold concentrated culture media improved control of LF (SC-EV + LysA2-PS + LF and SC-EV + LysA2-PS(50×) + LF in Figure 3); however, the improvement was not as substantial as with 100 µg/mL of intracellularly produced LysA2. The culture media from the empty vector *P. pastoris* provided no such benefit (SC-EV + PP-EV + LF in Figure 3), even when concentrated (SC-EV + PP-EV(50×) + LF in Figure 3). Since LP is insensitive to LysA2, the addition of purified, intracellularly-produced LysA2 was not effective at improving the reduced ethanol titer caused by LP (SC-EV + LP and SC-EV + LysA2-PI + LP in Figure 3). Based on this indication of lytic activity, the decision was made to attempt to express secreted LysA2 in *S. cerevisiae* despite the inability of a previous group to get a functional expression of LysA2 by *S. cerevisiae* [23].

### 3.4. S. cerevisiae Secreting LysA2 Restores Ethanol Yield in Mock Contamination of Fermentation

Unlike secreted LysA2 from *P. pastoris*, the putative LysA2 band secreted by *S. cerevisiae* was approximately 52 kDa (expected size with signal peptide) and another approximately 37 kDa band was observed. After several failed attempts to purify secreted LysA2, the two target bands were isolated from the protein gel and analyzed by LC/MS, following trypsin digestion. The smaller size LysA2 (37 kDa) could be a truncated LysA2 which has been cut by extracellular peptidase. Interestingly, glycosylation of LysA2 was not observed in *S. cerevisiae*.

**Figure 4:**
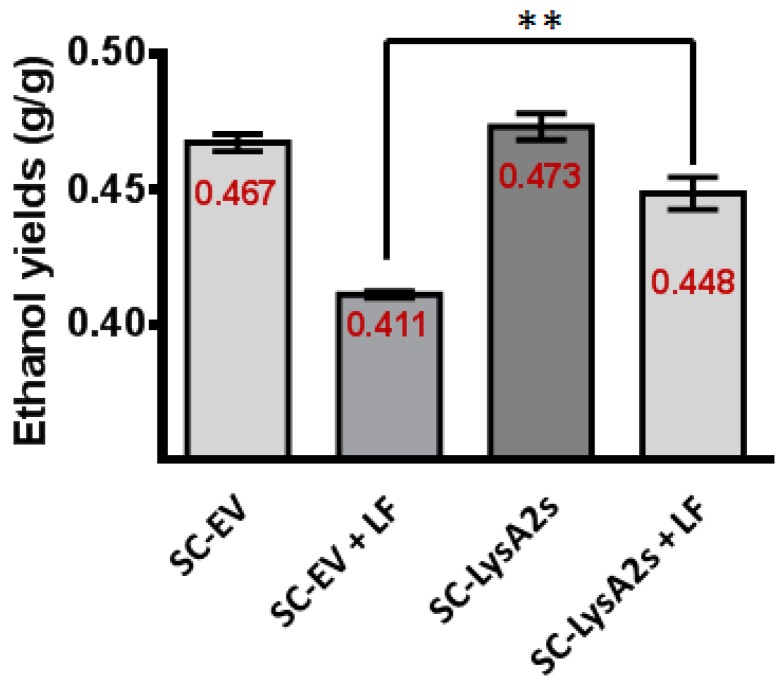
Impact of LysA2 secretion by *S. cerevisiae* on ethanol yield. In YPD60, the ethanol yield of *S. cerevisiae* secreting LysA2 (SC-LysA2s) showed 5% increase compared with *S. cerevisiae* with the empty vector (SC-EV) when L. fermentum (LF) is present. This result is significantly different. ** < 0.01.

*S. cerevisiae* containing the LysA2 secreting plasmid (SC-LysA2s), and the same strain with the vector lacking the LysA2 gene (SC-EV), were employed to test whether or not secreted LysA2 directly from *S. cerevisiae* can prevent contamination (Table 3). The fermentation profiles of both *S. cerevisiae* strains were similar when LF was not added. In the presence of LF, The LysA2 secreting *S. cerevisiae* strain fermented glucose and produced almost 29% more ethanol than the *S. cerevisiae* strain with the empty vector. It is likely that the *S. cerevisiae* strain with the empty vector failed to finish the fermentation due to inhibition by LF. With LF, the lactic acid concentrations in the culture medium were higher than the culture medium with the LysA2 secreting *S. cerevisiae*. As expected, the LysA2 secreting *S. cerevisiae* consumed glucose more rapidly, and increased ethanol yield by approximately 9%, while ethanol yield of the empty vector *S. cerevisiae* strain decreased in the presence of LF (Figure 4).

### 3.5. Mock Contamination with Sugarcane Juice

Next, we tested the ability of LysA2 secreting *S. cerevisiae* to prevent the impact of LF contamination in sugarcane juice (Table 4). In the absence of LF contamination, all strains had similar fermentation profiles. LF contamination reduced ethanol yield with the *S. cerevisiae* empty vector strain (SC-EV) by approximately 25% (0.48 to 0.31 g ethanol/g sugar) at the end of fermentation (48 h). The *S. cerevisiae* secreting LysA2 strain showed no improvement. Adding pulses of LysA2 (intracellularly produced by *P. pastoris*) during ethanol fermentation by *S. cerevisiae* increased ethanol yield by about 29%, compared to the fermentation with no added or secreted LysA2 (0.31 to 0.4 g ethanol/g sugar), and reduced lactic acid production. Integrating the LysA2 expression cassette into the *S. cerevisiae* genome increased the ethanol yield slightly in sugarcane juice, but not as much as the treatment with purified LysA2 (Table 4).

## 4. Discussion

In this study, our overall goal was to demonstrate that an endolysin secreting *S. cerevisiae* could be a useful tool for the bioethanol industry. Specifically, ethanol yield reduction mediated by bacterial contamination with engineered *S. cerevisiae* secreting the endolysin LysA2 during ethanol fermentation was alleviated. To date, bacteria secreting endolysins have been successfully engineered [37,38], and patent applications claim successful secretion by *P. pastoris* [39]; however, secretion by *S. cerevisiae* in ethanol fermentation has not been described.

### 4.1. Bacteria and Yeast-Based LysA2 Showed the Same Lytic Activity and Spectrum

Initial milestones critical to accomplishing this goal included: (1) endolysin produced intracellularly by yeast with activity comparable to bacterially produced endolysin, and (2) secretion of active endolysin by yeast. We employed *P. pastoris*, a yeast known for efficient protein expression and secretion [40], as a model system to confirm the lytic activity of yeast expressed LysA2. Isolated LysA2 from *P. pastoris* had similar lytic activity and lytic spectrum as those of LysA2 from *E. coli* (Figure 1 and Table 2).

In turbidity reduction assays, LysA2 from *P. pastoris* showed the highest lytic activity against the strains with the A4α peptidoglycan chemotype [41]. This is consistent with previous characterizations of LysA2 as an endopeptidase that hydrolyzes the bond between the terminal D-alanine and the D-aspartate, linking it to the neighboring peptide chain [25]. Interestingly, not all of strains with that cross linkage were equally sensitive (Table 2). One potential cause for this is interference from S-layer proteins, which are common in many *L. brevis* strains [42], but not confirmed in *L. casei* [43]. In addition, the cell wall binding domain may also be an important consideration to strain sensitivity [44,45,46]. As with most endolysins, a strain having the targeted linkage is not sufficient for sensitivity to LysA2 [24].

### 4.2. Secreted Lysa2 from P. Pastoris Successfully Restores Ethanol Productivity during Bacterial Contamination

A drawback of using yeast for secreting recombinant proteins is the prospect that the proteins will be glycosylated, which can result in impairment or loss of functionality. To investigate whether glycosylation would occur, and if it impacted LysA2 activity, we constructed a *P. pastoris* strain that secretes LysA2. After induction and suitable time for growth, protein expression in culture media was detectable, but the band was 70 kDa, i.e., much larger than the expected value, 42 kDa. Purification using the His6 tag of LysA2-PS failed, despite numerous attempts. The larger size and failure to bind to the resin is likely due to glycosylation of LysA2 at N or O linkages [47].

Although purification failed, we confirmed that LysA2 is secreted by the lytic activity of the culture supernatant and the lack of such activity in the culture supernatant of the *P. pastoris* empty vector strain. Concentration of the LysA2 containing supernatant resulted in increased lytic activity against *L. casei* (Figure 2), which is consistent with the dose response we saw with the purified, intracellularly produced LysA2 (Figure 1). Adding purified LysA2 and concentrated supernatant containing LysA2 improved ethanol yield during fermentations contaminated with *L. fermentum*. This species has been shown to reduce ethanol production in previous studies [3,11,48], and surveys of biofuel contaminants frequently identify *L. fermentum* [2,4,5]. These results are consistent with previous studies that found that uncontrolled LAB contamination decreases ethanol yield [3,13,48,49].

### 4.3. S. cerevisiae Secreting LysA2 is Effective Against Bacterial Contamination in YPD Medium

Compared with *P. pastoris* strains, *S. cerevisiae* strains may hyper-glycosylate proteins, and add terminal mannoses in a bond which may be allergenic [50]. In addition, increasing secretion yield from *S. cerevisiae* typically requires complex optimization steps [51]. Despite these challenges, because *S. cerevisiae* is the most commonly used species for bioethanol production, we wanted to study the question of whether *S. cerevisiae* D452-2 with the LysA2 secreting plasmid could mitigate the impact of LAB contamination. In simulated contaminations in YPD60, the LysA2 secreting *S. cerevisiae* strain provided higher ethanol productivity and yield, compared to the *S. cerevisiae* empty vector strain when challenged with *L. fermentum* (Table 4 and Figure 4). This result was consistent with our experiment controlling contaminants with *P. pastoris* produced LysA2 (Figure 3). The ethanol productivity and yield data demonstrate that, in our system, secreted LysA2 from *S. cerevisiae* could increase ethanol productivity by inhibiting bacterial growth. However, even with LysA2 secretion, the contamination reduced ethanol yield compared to the uncontaminated cultures suggests that improvements in LysA2 secretion could be beneficial.

### 4.4. S. cerevisiae Secreting LysA2 Fails to Protect Against Bacterial Contamination in Sugarcane Juice

To test the impact of LysA2 on the inhibition of bacterial growth in media similar to bioethanol production in Brazil, contaminations using diluted sugarcane juice as the fermentation substrate were performed. Sugarcane juice, a critical feedstock for the ethanol fermentation in Brazil [2,52], contains primarily sucrose, along with lesser amounts of other reducing sugars and organic material and minerals which vary between batches [53]. Unfortunately, in sugarcane juice, secreting LysA2 by *S. cerevisiae* did not provide a notable improvement of ethanol yield when challenged with LF contamination. We subsequently moved the LysA2 expression cassette into the *S. cerevisiae* genome, to determine if plasmid instability was an issue. The genome integrated LysA2 strain did show a slight increase of ethanol production and yield. However, adding pulses of 50 μg/mL of intracellularly produced LysA2 from *P. pastoris* improved ethanol production, confirming that LysA2 is functional in sugarcane juice. *S. cerevisiae* D452-2 grew very poorly in sugarcane juice (48 h to consume 60 g sucrose; Table 4), especially compared to its growth in YPD (24 h to consume 62 g glucose; Table 3). This indicates that the *S. cerevisiae* secretion process for LysA2 is not enough to ameliorate bacterial contamination; hence, improvement of the expression level is required. Multicopy integration of the LysA2 gene into the *S. cerevisiae* genome [2,54] could result in improved contaminant control in sucrose fermentations. Alternatively, using an inducible promoter, instead of expressing the protein constitutively (which may place undue stress on the host), may improve contamination control with the secreted LysA2. Regardless, we recommend that future work focus on improving the lytic spectrum of LysA2, and/or on identifying new endolysins with a suitable lytic spectrum, so that more of the problematic LABs are controlled by this treatment.

## 5. Conclusions

Both *P. pastoris* and *S. cerevisiae* are capable of producing the endolysin LysA2 in its active form. The intracellularly produced LysA2 from *P. pastoris* showed activity comparable to bacterially produced LysA2. LAB with the A4α peptidoglycan chemotype (L-Lys-D-Asp crosslinkage) were the most sensitive to LysA2, though a few from that chemotype were insensitive. In our experiments, purified *Pichia*-expressed LysA2 successfully improved ethanol productivity and yields in glucose (YPD60) and sucrose-based (sugarcane juice) ethanol fermentation in the presence of a susceptible LAB contaminant. LysA2 secreting *S. cerevisiae* was also able to control bacterial contamination during fermentation in YPD60, but not in sugar cane juice. Secretion of LysA2 by the fermenting yeast, or adding it in purified form, is a promising alternative tool for controlling LAB contamination during ethanol fermentation. In an optimized process, both options could supplement or replace the currently used antibiotics, or the acidic washing step which inhibits yeast productivity [35,52,55].

## Figures and Tables

**Table 1:** Bacterial and yeast host strains used in this study.

Strains	Description
*Escherichia coli*	BL21 (DE3)
*Escherichia coli*	BL21 (DE3) containing pRSETA_LysA2
*Pichia pastoris*	GS115
*Pichia pastoris*	GS115 containing pPICZAa
*Pichia pastoris*	GS115 containing pPICZA_LysA2
*Pichia pastoris*	GS115 containing pPICZAa_LysA2
*Saccharomyces cerevisiae*	D452-2
*Saccharomyces cerevisiae*	D452-2 containing pRS423_LysA2
*Saccharomyces cerevisiae*	D452-2 containing pITY3_LysA2 ^1^
*Saccharomyces cerevisiae*	D452-2 empty vector

^1^ Chromosomal insertion.

**Table 2:** Lytic activity of LysA2 produced intracellularly by *P. pastoris* against 13 lactic acid bacteria representative of common biofuel contaminants.

Bacterial Contaminants	ATCC #	Peptidoglycan Chemotype	Relative Activity ^1^
*Aerococcus viridans*	11563	A1α direct	−
*Lactobacillus plantarum*	14917	A1γ mesoDpm	−
*Lactobacillus casei*	393	A4α L-Lys-D-Asp	Positive control ^2^
*Enterococcus faecium*	6057	A4α L-Lys-D-Asp	++
*Enterococcus gallinarum*	49573	A4α L-Lys-D-Asp	++
*Lactobacillus brevis*	14869	A4α L-Lys-D-Asp	−
*Lactobacillus delbrueckii*	9649	A4α L-Lys-D-Asp	+++
*Lactobacillus paracasei*	25598	A4α L-Lys-D-Asp	++++
*Lactobacillus rhamnosus*	53103	A4α L-Lys-D-Asp	+
*Lactococcus lactis*	19257	A4α L-Lys-D-Asp	+
*Pediococcus acidilactici*	NA	A4α L-Lys-D-Asp	−
*Pediococcus damnosus*	29358	A4α L-Lys-D-Asp	−
*Lactobacillus fermentum*	9338	A4β L-Orn-D-Asp	++

^1^ Relative activity measured by turbidity reduction in the presence of 100 nM LysA2 after 1 h incubation as described in the methods section; (−) = 0–10%, (+) = 11–25%, (++) = 26–50%, (+++) = 51–75%, (++++) = 76–100%. ^2^ *L. casei* used as the positive control with all data relative to the turbidity reduction of this strain. NA = not available.

**Table 3:** Fermentation profile of SC-EV ^1^ and SC-LysA2s ^2^ in simulated contamination.

Contaminants	Yeast Strain	n	Glucose Consumption (g/L ± SEM)				Lactic Acid (g/L ± SEM)				Ethanol (g/L ± SEM)			
			0 h	6 h	12 h	24 h	0 h	6 h	12 h	24 h	0 h	6 h	12 h	24 h
**None**	**SC-EV ^1^**	**3**	0.0 ± 0.0	2.6 ± 0.3	13.7 ± 0.3	62 ± 0.4	ND	ND	ND	ND	0.0 ± 0.0	0.6 ± 0.1	6.1 ± 0.1	26.8 ± 1.8
	**SC-LysA2s ^2^**	**3**	0.0 ± 0.0	3.2 ± 0.0	15.1 ± 0.5	62 ± 0.0	ND	ND	ND	ND	0.0 ± 0.0	0.8 ± 0.3	8.0 ± 0.2	27.3 ± 0.2
***L. fermentum***	**SC-EV ^1^**	**3**	0.0 ± 0.0	4.7 ± 0.0	17.1 ± 0.3	50.6 ± 0.2	0.0 ± 0.0	0.7 ± 0.0	1.8 ± 0.0	3.4 ± 0.0	0.0 ± 0.0	0.8 ± 0.0	6.2 ± 0.3	20.8 ± 0.1
	**SC-LysA2s ^2^**	**3**	0.0 ± 0.0	5.1 ± 0.8	19.3 ± 0.4	62.0 ± 0.0	0.0 ± 0.0	0.6 ± 0.0	1.2 ± 0.0	2.5 ± 0.0	0.0 ± 0.0	1.1 ± 0.3	7.1 ± 0.2	26.8 ± 0.2

^1^*S. cerevisiae* with empty vector; ^2^ *S. cerevisiae* that contains LysA2 secretion vector; ND = not determined.

**Table 4:** Fermentation profile of SC-EV ^1^, SC-LysA2s ^2^, SC-LysA2s* ^3^ and purified LysA2 ^4^ with *L. fermentum* in sugarcane.

Contaminant	Yeast Strain	n	Ethanol Production (g/L ± SEM)				Lactic Acid (g/L ± SEM)				Ethanol yield (g/g ± SEM)			
			0 h	12 h	24 h	48 h	0 h	12 h	24 h	48 h	0 h	12 h	24 h	48 h
**None**	**SC-EV ^1^**	**3**	0.0 ± 0.0	6.8 ± 0.5	15.5 ± 0.5	28.4 ± 0.1	ND	ND	ND	ND	0	0.46	0.48	0.48
	**SC-LysA2s ^2^**	**3**	0.0 ± 0.0	6.9 ± 0.1	15.4 ± 0.2	27.8 ± 0.0	ND	ND	ND	ND	0	0.45	0.48	0.48
	SC-LysA2s* ^3^	**3**	0.0 ± 0.0	6.9 ± 0.1	16.4 ± 0.3	28.8 ± 0.2	ND	ND	ND	ND	0	0.45	0.48	0.48
	**SC-EV/LysA2-PI ^4^**	**3**	0.0 ± 0.0	6.7 ± 0.3	16.2 ± 0.2	27.8 ± 0.2	ND	ND	ND	ND	0	0.46	0.48	0.48
***L. fermentum***	**SC-EV ^1^**	**3**	0.0 ± 0.0	6.4 ± 0.1	13.4 ± 0.4	18.6 ± 0.4	0.0 ± 0.0	1.1 ± 0.0	2.8 ± 0.0	3.5 ± 0.0	0	0.25	0.27	0.31
	**SC-LysA2s ^2^**	**3**	0.0 ± 0.0	6.6 ± 0.2	13.2 ± 0.2	18.4 ± 0.0	0.0 ± 0.0	1.3 ± 0.0	2.6 ± 0.0	3.4 ± 0.0	0	0.24	0.27	0.32
	SC-LysA2s* ^3^	**3**	0.0 ± 0.0	6.2 ± 0.2	15.5 ± 0.2	20.3 ± 0.0	0.0 ± 0.0	0.6 ± 0.0	2.2 ± 0.0	3.1 ± 0.0	0	0.24	0.30	0.35
	**SC-EV/LysA2-PI ^4^**	**3**	0.0 ± 0.0	6.0 ± 0.2	18.7 ± 0.2	23.6 ± 0.3	0.0 ± 0.0	0.4 ± 0.0	1.2 ± 0.0	2.2 ± 0.0	0	0.41	0.39	0.4

^1^*S. cerevisiae* with empty vector; ^2^ *S. cerevisiae* that contains LysA2 secretion vector; ^3^ *S. cerevisiae* that has the LysA2 secretion vector integrated into the yeast genome; ^4^ *S. cerevisiae* with empty vector supplemented with purified, intracellularly produced LysA2 from *P. pastoris* (LysA2-PI); ND = not determined.

## Supplementary Materials

The following are available online at http://www.mdpi.com/1999-4915/10/6/281/s1, Figure S1: Constructed vector maps for LysA2 expression in this study. Figure S2: The LysA2 secretion by integrated *LysA2* gene into *S. cerevisiae*.
