# Supplementary material for: Yeast Derived LysA2 Can Control Bacterial Contamination in Ethanol Fermentation
Source: Viruses. 2018 May 24;10(6):281. doi: 10.3390/v10060281 (PMC6024572; doi:10.3390/v10060281)
Supplement: Supplementary file 1 [file viruses-10-00281-s001.pdf]

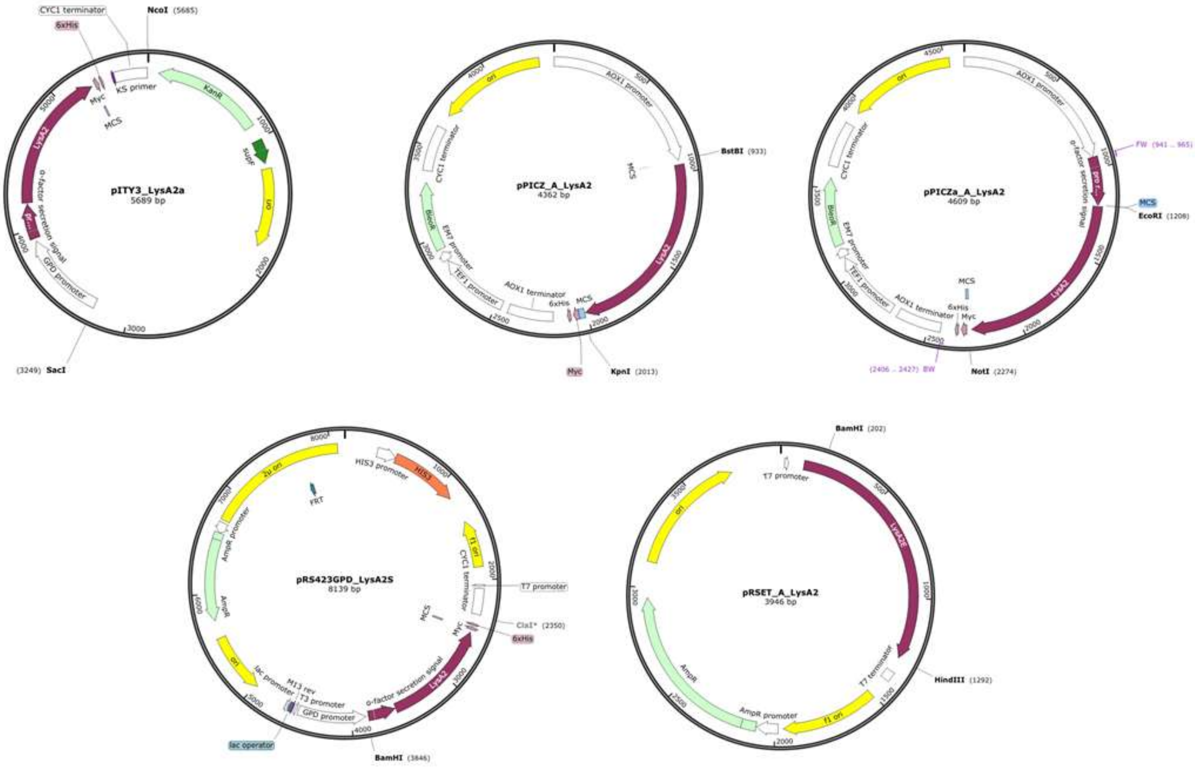

Figure S1. Constructed vector maps for LysA2 expression in this study.

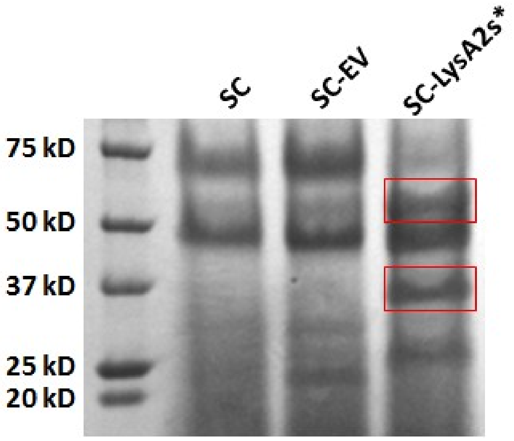

Figure S2. The LysA2 secretion by integrated *LysA2* gene into *S. cerevesiae*. Secreted LysA2 from *S. cerevisiae* D452-2. Unlike secreted LysA2 from *P. pastoris*, glycosylation was not observed.
